# Mycoparasite *Hypomyces odoratus* infests *Agaricus xanthodermus* fruiting bodies in nature

**DOI:** 10.1186/s13568-020-01085-5

**Published:** 2020-08-13

**Authors:** Kiran Lakkireddy, Weeradej Khonsuntia, Ursula Kües

**Affiliations:** 1 Department of Molecular Wood Biotechnology and Technical Mycology, Büsgen-Institute, Georg-August-University, Göttingen, Germany; 2 Center for Molecular Biosciences (GZMB), Georg-August-University, Göttingen, Germany; 3 Present Address: Faculty of Agricultural Technology, Rajabhat Mahasarakham University, Mueang Maha Sarakham District, Maha Sarakham Thailand

**Keywords:** Mycopathogen, *Hypomyces*, *Agaricus*, Mushrooms, Conidiation, Microsclerotia

## Abstract

Mycopathogens are serious threats to the crops in commercial mushroom cultivations. In contrast, little is yet known on their occurrence and behaviour in nature. Cobweb infections by a conidiogenous *Cladobotryum*-type fungus identified by morphology and ITS sequences as *Hypomyces odoratus* were observed in the year 2015 on primordia and young and mature fruiting bodies of *Agaricus xanthodermus* in the wild. Progress in development and morphologies of fruiting bodies were affected by the infections. Infested structures aged and decayed prematurely. The mycoparasites tended by mycelial growth from the surroundings to infect healthy fungal structures. They entered from the base of the stipes to grow upwards and eventually also onto lamellae and caps. Isolated *H. odoratus* strains from a diseased standing mushroom, from a decaying overturned mushroom stipe and from rotting plant material infected mushrooms of different species of the genus *Agaricus* while *Pleurotus ostreatus* fruiting bodies were largely resistant. Growing and grown *A.* *xanthodermus* and *P. ostreatus* mycelium showed degrees of resistance against the mycopathogen, in contrast to mycelium of *Coprinopsis cinerea*. Mycelial morphological characteristics (colonies, conidiophores and conidia, chlamydospores, microsclerotia, pulvinate stroma) and variations of five different *H.* *odoratus* isolates are presented. In pH-dependent manner, *H.* *odoratus* strains stained growth media by pigment production yellow (acidic pH range) or pinkish-red (neutral to slightly alkaline pH range).

## Introduction

Commercially cultivated mushrooms can be attacked by distinct mycoparasites such as the edible *Agaricus bisporus* by the ascomycetes *Lecanicillium fungicola*, *Mycogone perniciosa* (teleomorph *Hypomyces perniciosus*), and *Cladobotryum dendroides* (teleomorph *Hypomyces rosellus*) which cause dry bubble, wet bubble and cobweb disease, respectively (Largeteau and Savoie 2010; Berendsen et al. 2010; Carrasco et al. 2017). Such infections can result in severe crop losses, particularly in later flushes, if hygienic standards during cultivation are not high. Infections might originate from contaminated soil or spawn and the fungi might be introduced into mushroom casing in the form of spores or mycelium (Adie et al. 2006; Soković and Van Griensven 2006; Szumigaj-Tarnowska et al. 2015; Carrasco et al. 2017).

*Lecanicillium fungicola* not only infects the generative stage of *A. bisporus* but at all phases of fruiting body development (North and Wuest 1993; Calonje et al. 2000; Bernardo et al. 2004; Largeteau et al. 2007; Nunes et al. 2017). Depending on the developmental stage that becomes infected, disease symptoms range from totally undifferentiated spherical masses formed together by mycelia of host and pathogen (“dry bubble”), over partial disruption of stipe and cap tissues resulting in stipe deformations (“stipe blowout”) to small necrotic lesions in the cap (“spotty cap”) (North and Wuest 1993; Soler-Rivas et al. 2000; Largeteau et al. 2007, Largeteau and Savoie 2010; Bailey et al. 2013). Early infection of fruiting body initials by *M. perniciosa* also leads to the formation of undifferentiated hyphal masses (“sclerodermoid mushrooms”). These “wet bubbles” are first white and spongy. Then, they turn brownish and may be covered by amber-coloured liquid excretions. Mushroom deformations and cap spotting result from infections at later developmental stages (Fletcher et al. 1995; Umar and Van Griensven 1999; Umar et al. 2000; Glamoclija et al. 2008; Kouser and Shah 2013; Zhang et al. 2017). The soil inhabiting *C. dendroides* covers all stages of fruiting bodies in form of coarse white mycelium (“cobweb”) under massive conidiospore production. Overgrown mushrooms eventually rot and collapse. Further symptoms linked to cobweb disease are brown spotting on caps instigated by germinating spores (Bhatt and Singh 2002; Potočnik 2006; Parrag et al. 2014; Carrasco et al. 2017). In recent time, other *Cladobotryum* species (mainly *C.* *mycophilum*, teleomorph *Hypomyces odoratus*; *C. varium*, teleomorph *Hypomyces aurantius*) have more often been reported to cause cob-web diseases including cap spotting and patching on *A. bisporus* (McKay et al. 1999; Grogan and Gaze 2000; Back et al. 2010, 2012b; Lee et al. 2011; Sharma et al. 2015; Carrasco et al. 2016, 2017; Chakwiya et al. 2019). According to McKay et al. (1999), Grogan (2006) and Tamm and Põldmaa (2013), when *H.* *odoratus* occurs in mushroom farms, it is quite often misidentified under the name *H. rosellus*. The sexual fruiting bodies (perithezia) cannot easily be differentiated morphologically between the species unlike their conidiophores with the asexual conidia (Rogerson and Samuels 1993, 1994). Asexual strain features together with molecular data are therefore used to define species (Kirschner et al. 2007; Põldmaa 2011; Tamm and Põldmaa 2013; Gea et al. 2019).

The different mycopathogens are not restricted to *A. bisporus* but may affect also other commercially cultivated species. Incidences of *L. fungicola* disease were reported for other *Agaricus* species (Gea et al. 2003) and *Pleurotus ostreatus* (Marlowe and Romaine 1982). *M. perniciosa* is shown to also infect *Pleurotus eryngii* and *Pleurotus nebrodensis* as well as *Volvariella volvaceae*, with the result of fruiting body malformations (Sisto et al. 1997; Sharma and Kumar 2000; Carrasco et al. 2017). Aggressive cobweb infections by *Cladobotryum* species were described for cultured *Calocybe indica* (Sharma et al. 2015), *Coprinus comatus* (Wang et al. 2015), *Flammulina velutipes* (Kim et al. 1999; Back et al. 2012b), *Ganoderma tsugae* (Kirschner et al. 2007), *Hypsizygus marmoreus* (Back et al. 2012a, b, 2015), *Pleurotus sajor*-*caju* (Sharma et al. 2015), *P. eryngii* (Kim et al. 1998, 2014; Gea et al. 2011, 2016, 2017; Back et al. 2012b), and *P. ostreatus* (Pérez-Silva and Guevara 1999; Gea et al. 2019).

While attention is paid on pathogen infections in commercial mushroom cultures due to the high economic interest, infection events observed in nature are scattered and usually not deeply described. In nature, an association with basidiomycete fruiting bodies and verticillium-like anamorphs (conidiophores are verticillate with whorls of few to several phialides which give rise to the phialoconidia) can help to identify potential mycopathogens (Gray and Morgan-Jones 1980; Zare and Gams 2008; Rogerson and Samuels 1989, 1993, 1994; Põldmaa and Samuels 1999; Põldmaa 2003; Tamm and Põldmaa 2013; Chakwiya et al. 2019). From the wild, *L. fungicola* has been isolated from fruiting bodies of *Agaricales* (e.g. *Marasmiellus ramealis*, *Hypholoma capnoides* and *Laccaria laccata*) and of decaying samples of *Thelephora terrestris* from the *Thelephorales*. *Lecanicillium flaccidum* from the same species complex was obtained from basidiocarps of *Coltricia perennis* of the *Hymenochaetales* and of *Gomphidius glutinosus* from the *Boletales*, and of decaying samples of *Russula nigricans* of the *Russulales* (Zare and Gams 2008). Incidences of *Hypomyces*/*Cladobotryum* infections appear to be more common. *C. dendroides* and *C. mycophilum* have a broad host range and have been isolated from mushrooms of varied species of *Agaricales*, *Boletales*, *Hymenochaetales*, *Polyporales*, *Russulales*, *Telephorales* and others. However, there are several more mycopathogens between the paraphyletic *Hypomyces*/*Cladobotryum* species group, several of which are producing yellow to red-coloured pigments and some of which have a more restricted host range (Gray and Morgan-Jones 1980; Sohi and Upadhyay 1986; Rogerson and Samuels 1989, 1993, 1994; Helfer 1991; Põldmaa and Samuels 1999; Douhan and Rizzo 2003; Põldmaa 2003; Valdez and Douhan 2012; Tamm and Põldmaa 2013; Marzuko et al. 2015; Wang et al. 2015; Zare and Gams 2016). In particular, orange-red lobster mushrooms are fruiting bodies of *Russula*, *Lactarius* and *Lactifluus* species from the *Russulales* which are infested by staining *Hypomyces lactifluorum* and are collected and commercially marketed as culinary delicacy in Mexico and Northern America (Laperriere et al. 2018).

In this report, we describe our observations on infestations of *Agaricus xanthodermus* fruiting structures in nature with strongly sporulating ascomycetous mycopathogens. We isolated mycopathogenic strains from infested material and describe their morphology and molecular identity with ITS sequences as *H. odoratus*/*C. mycophilum*. Furthermore, we performed infection studies with vegetative mycelium and fruiting structures of different basidiomycetous species.

## Materials and methods

### Mushroom observations, collection and fungal strain isolation

Mushrooms of *A.* *xanthodermus* growing underneath a *Pseudotsuga menzii* tree on the north side next to building Büsgenweg 5 of the Faculty of Forest Sciences and Forest Ecology (latitude 41.55933; longitude 9.95722) on the grounds of the North Campus of the University of Göttingen were usually observed and photographed at noon (at about 13 to 14 o’clock). Climate data (temperature and humidity) were routinely collected on the grounds through a hygro-thermo transmitter (Adolf Thies GmbH & Co. KG, Thies Clima, Göttingen, Germany). Mushrooms were identified by morphology using Breitenbach and Kränzlin (1995).

Crippled and decaying mushrooms were collected as well as rotting grass/moss samples with obvious white fungal mycelium. The samples were directly brought to a classroom laboratory and photographed by an IXUS 115 HS digital camera (Canon, Krefeld, Germany). For enlarged views, a M205 FA stereomicroscope with an integrated CF420 camera was used and the Leica Application Suite v3.8 software (Leica, Wetzlar, Germany). Samples of infesting mycelium from the cap of a crippled mushroom and mycelial samples of isolated cultures were observed with an Axioplan 2 imaging microscope (Carl Zeiss, Göttingen, Germany) equipped with a Soft Imaging System ColorView II digital camera. Digital photos taken were processed with the Soft Imaging System analySIS software (EMSIS, Münster, Germany). Size parameters were measured with the Arbitrary Distance function of the program and Excel (Microsoft, Redmond, WA) was used for calculations.

To isolate the basidiomycete, small mycelial samples were aseptically taken from the inner stipe regions of a healthy mushroom, and tissues were transferred onto MEA (2% malt extract, 1% agar; initial pH 5.0) plates with added antibiotics (ampicillin 100 µg/ml, kanamycin 50 µg/ml, tetracycline 10 µg/ml, chloramphenicol 20 µg/ml and streptomycin 100 µg/ml) as described formerly in Badalyan et al. (2011). To isolate the potential mycopathogens, foreign mycelia were taken from outer infested stipe and cap regions as well as from a grass/moss sample and transferred onto MEA plates supplemented with antibiotics. Plates were incubated at room temperature (RT) in the classroom. Growing mycelial samples were transferred for strain isolation and colony observations onto fresh MEA and YMG/T (0.4% yeast extract, 1% malt extract, 0.4% glucose, 0.001% tryptophan, 1% agar; Granado et al. 1997; initial pH 6) for growth at RT. Plastic Petri dishes (9 cm in Ø) with vents were used. Yeast extract (LP0021) and malt extract (L39) were from Oxoid (Basingstroke, UK), agar (Nr. 11396) from Serva (Heidelberg, Germany).

The isolated dikaryotic mycelium of *A.* *xanthodermus* (strain KKRL1) and the five different mycopathogen isolates (AscoA1, AscoB1, AscoC1, AscoD1, AscoE1) of this study were deposited in the DSMZ (Deutsche Sammlung von Mikroorganismen und Zellkulturen GmbH) strain collection in Braunschweig (Germany) under Catalog numbers DSM 111245 (KKRL1) to DSM 111250 (AscoA1 to AscoE1), respectively.

### Colony characterisation

Cultures were grown at RT if not otherwise stated. Cultures were photographed with the IXUS 115 HS digital camera. pHs of culture medium were estimated with pH indicator strips which were dipped into squeezed agar pieces cut out from fresh and from mycelium overgrown medium. Mycelial samples with conidiophores, conidia or chlamydospores were observed under a Zeiss Axioplan 2 imaging microscope, digital photos were taken and size parameters measured with the analySIS software as described above. Diameters of chlamydospores as of microsclerotia and dense mycelial patches were measured crosswise in two directions and averages were calculated from all data. White mycelial patches were analysed in digital photos of complete cultures and microsclerotia using colony views of older cultures with collapsed aerial mycelium as photographed under the M205 FA stereomicroscope. Conidia from fully grown whole cultures were harvested from the culture surfaces as described in Kertesz-Chaloupková et al. (1998), spores attached to the lids of Petri dishes were washed off with sterile water and added to the spores harvested from the colony surfaces and total spores were counted using a hematocytometer.

### ITS sequencing

Genomic DNA was isolated from mushroom samples taken from outside and from mycelium in culture (Zolan and Pukkila 1986). ITS sequences of basidiomycetes were PCR-amplified with primers ITS1 (TCCGTAGGTGAACCTGCGG) and ITS4 (TCCTCCGCTTATTGATATGC) (White et al. 1990) and of ascomycetes with primers ITS-1* (TCCGTTGGTGAACCAGCGG) (Waalwijk et al. 1996) and ITS4 and analyzed as described before (Naumann et al. 2007). Gene sequences were deposited in GenBank under the Accession numbers KX098646-KX098654.

### Mushroom infestation tests

Commercial mushrooms of *A. bisporus* (cap Ø 3.7 to 5.7 cm) and *P. ostreatus* (cap width between 2.4 and 6.7 cm) were purchased from a local supermarket. *A. bisporus* fruiting bodies were longitudinally cut into halves and transferred into sterile crystal dishes (18.5 cm in Ø, 4.5 cm in height) with the cut side alternatively positioned to the top or to the bottom of the dish. Other *A. bisporus* mushrooms were used in whole in erect condition. *P. ostreatus* fruiting bodies were used either in whole or as halves in upside-top (lamellae oriented down) and in upside-down position (lamellae oriented to the top). Non-injured caps or cuts of caps or cut or non-cut sides of stipes of the fruiting bodies of *A.* *bisporus* and either caps or stipes of *P. ostreatus* were infested with small freshly grown MEA agar pieces of mycelial isolates, the crystal dishes were closed by their lids and incubated at RT. Every 12 to 24 h, mushrooms were inspected and photographed. For every isolate, at least 35 mushroom samples of *A. bisporus* and 25 mushroom samples of *P.* *ostreatus* were tested in at least 4 rounds of experiments.

Further, *Agaricus* mushrooms collected in September 2015 from the wild in other places in Göttingen-Weende/-Nordstadt were transferred into sterile glass jars and infested either on the cap or at the bottom of the stipe by small MEA agar pieces with freshly grown mycelial samples. Mushroom identities were determined by morphological means (Breitenbach and Kränzlin 1995) and ITS sequencing as *A. xanthodermus* (KX098653) and *Agaricus* sp. section *Arvenses* (KX098654).

### Culture infestation tests

Mycelial cultures of *Coprinopsis cinerea* strain AmutBmut (*A43mut*, *B43mut*, *pab1*-*1*; Kertesz-Chaloupková et al. 1998), *P. ostreatus* monokaryon Pc9 (CECT20311), and of the isolated dikaryon KKRL1 of *A. xanthodermus* were prepared by inoculating one or two small freshly grown mycelial samples in the middle or at equal distances distributed on MEA or YMG/T plates and incubating them for vegetative growth at 37 °C (*C.* *cinerea* for subsequent grown mycelial challenge tests) or room temperature (RT, about 22 °C, used for other species in all grown mycelial challenge tests). Once a basidiomycete mycelium was fully established, a culture was challenged with two ca. 1 × 1 mm small inocula of freshly grown MEA agar pieces of a mycelial isolate to be tested by placing them onto the already grown basidiomycete mycelium 2 cm apart from the basidiomycete inoculum. The dual cultures were further incubated at RT and observed on daily basis for at least 20 days and in some instances for up to 2 months. Plates were photographed by an IXUS 115 HS digital camera. Five (*A.* *xanthodermus*) to six repeats (others) with two to three plates each were followed up per strain combination and MEA or YMG/T medium. Mycelial samples were observed under a Zeiss Axioplan 2 imaging microscope (Carl Zeiss, Göttingen, Germany).

In other sets of experiments (mycelial confrontation tests), basidiomycetes were inoculated on MEA or YMG/T medium 1.5 cm apart from the edge of a Petri dish (and pregrown when needed; see “Results” section), and the mycelial test strains 1.5 cm apart from the edge of the opposite side of the Petri dish. All plates were incubated at RT and observed for about a month and more after they were fully overgrown by the two mycelia. Five (*A.* *xanthodermus*) to six repeats (others) with two to three plates each were followed up per combination on MEA medium or YMG/T medium. Plates were regularly observed and photographed by an IXUS 115 HS digital camera and under a Zeiss Stemi 2000-C Binocular (Carl Zeiss, Göttingen, Germany). Presence of conidiophores and -spores of test isolates and hyphae of basidiomycetes were followed up by observing small mycelial samples from confrontation zones under a Zeiss Axioplan 2 imaging microscope.

## Results

### Mushroom development of *Agaricus xanthodermus* in nature

**Fig. 1:**
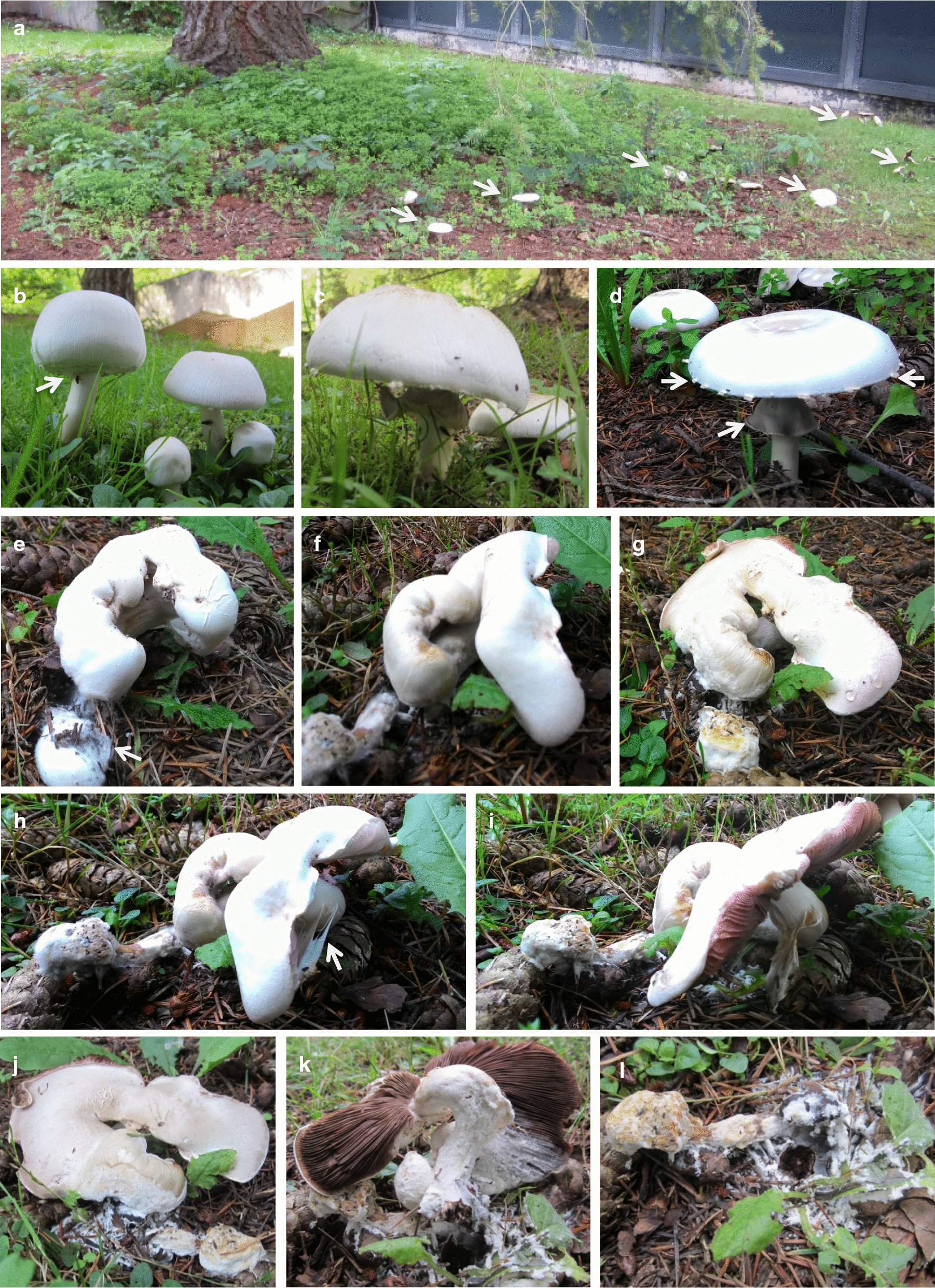
*Agaricus xanthodermus* fruiting bodies. **a** Mushrooms (marked by arrows) underneath a *Pseudotsuga menziesii* tree on the 3rd of September 2015. **b** Drum-stick-like young mushrooms: the left one is grown to full size (the arrow points to the partial veil underneath the cap). **c** Mushroom opening and **d** fully opened mushroom with vestiges of the partial veil at the edge of the opened cap and a skirt-like annulus around the stipe (marked by arrows). **e**–**l** Diseased crippled young mushroom with split stipe and cap and an infested primordium partially covered by a fluffy foreign mycelium (marked by an arrow) on 1st of September 2015 at the day of detection (**e**), 1 day after (**f**, **h**; the arrow points to partial veil still attached to cap tissues), 2 days after (**g**, **i**; note the pinkish still healthy lamellae in **i**) and 3 days after (**j**), when the mushroom was harvested (**k**; note the now brown colour of the lamellae and the white foreign mycelium which covers the crippled stipe and grows onto the lamellae). After harvest, white mycelium was seen spread over the needle and cone litter layer, the decayed primordium and a cone from the Douglas fir underneath (**l**)

Since 2012, we observed every year but in 2018 and 2019 as 2 years with very dry hot summers that multiple white fruiting bodies of an *Agaricus* species appeared singly or in small loose groups variably in the months June to November in the thick layer of needle and cone litter underneath a *P.* *menziesii* (Douglas fir) tree and in the nearby grass of the surrounding meadow on the North Campus of Göttingen University (Fig. 1a). Initially, we noticed the conspicuous mushrooms either in still closed or in already opened conditions. Later with better attention we also saw smaller primordia (< 1 cm in Ø) emerging through the soil from broken ground. Mushroom production appeared to correlate with 1 to 3 prior days of high humidity triggered by good rainfall (about 80% and 95% humidity at days and nights) when temperatures reduced with the rainfall by about 5 to highest 10° from prior day time temperature values which were between 18 and to up to 30 °C at former warmer days (the actual temperatures depended on the time of the year). Spherical primordia were observed aboveground 2 to 4 days after the inducing days of high humidity and reduced temperature, still closed mushrooms with lengthened stipes (“drum-sticks”) 4 to 8 days and opened mushrooms 10 to over 20 days after the rainfalls, when the days following induction were sunny and again warmer in temperature by an increase of 2 to 5° and when humidity values differed between night (ca. 80–95% humidity) and day periods (ca. 60–85% humidity). Fruiting body development continued usually in a temperature range from 15 to slightly above 20 °C. However, depending on the month there were also exceptional days encountered in later fruiting body development with temperatures up to 30 °C.

**Fig. 2:**
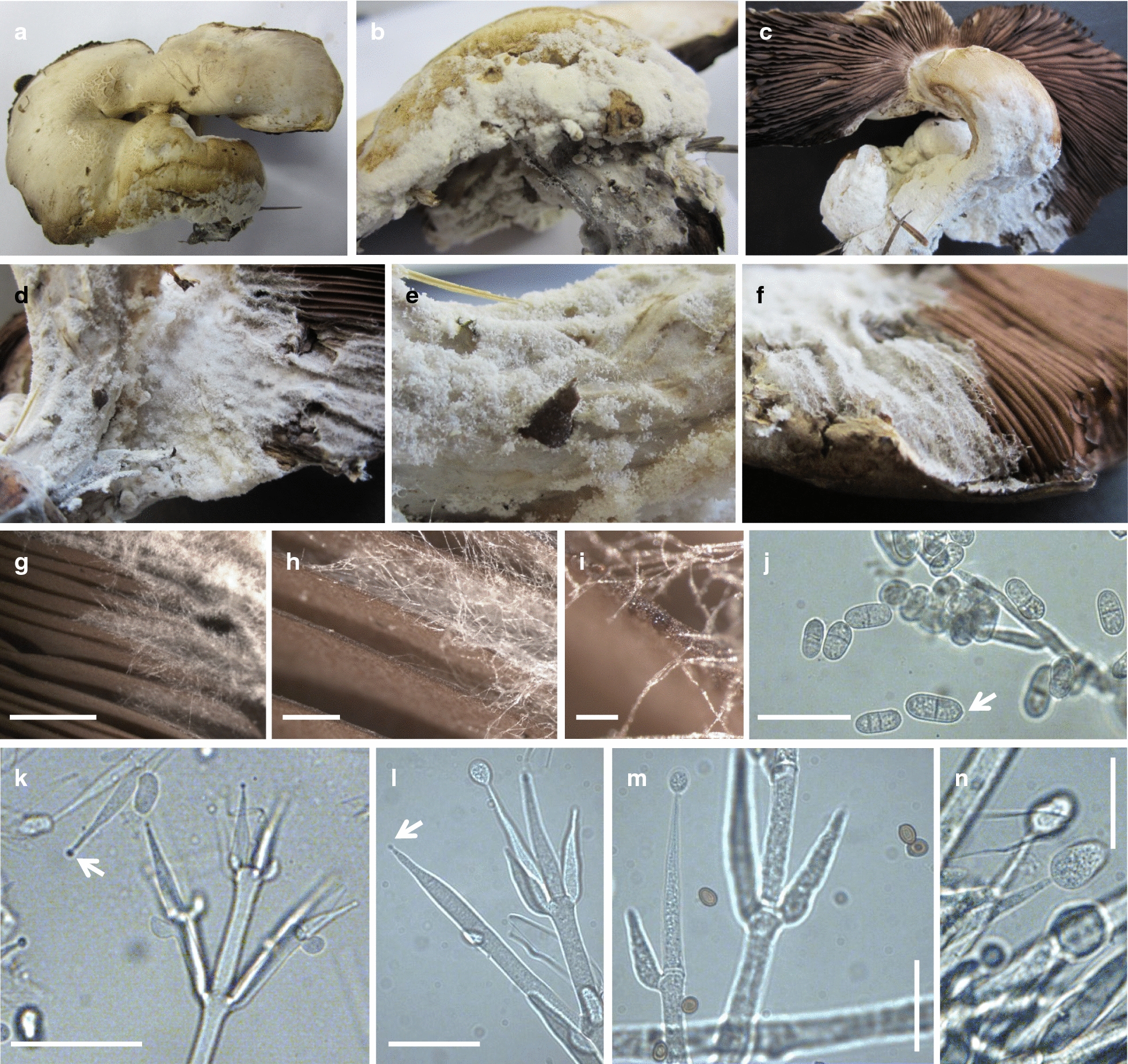
Diseased crippled *Agaricus xanthodermus* fruiting body. **a**, **b** The upper surface of the cap, **c**, **d** the surface of the bended stipe, and **e**, **f** parts of the lamellae are overgrown by foreign mycelium with conidiophores visible as white flocks in the aerial mycelium (**d**). **g**–**i** For infestation of the lamellae, the foreign mycelium grew first over their edges, with conidiophore production starting about 6 to 7 mm behind the growth front (**g**; see white flocks at the right side of the photo). **j**–**n** Conidia and conidiophores in mycelial samples taken for microscopy from the lamellae. Note the blastic generation of conidiospores at the tips of phialides (arrows in **k**–**l**) and dehiscence scars (basal hilum) at the spores (see arrow in **j**) and also the small brown basidiospores of the host (**m**). Sizes bars correspond to 5 mm (**g**), 1 mm (**h**), 200 µm (**i**), 50 µm (**k**) and 20 µm (**j**, **l**–**n**)

We observed round ball-like primordia (about 1.5–2 cm in Ø) on the floor and closed young white mushrooms that had a drum-stick shape and were generated from the spherical primordia by stipe growth and increase in cap size. Growth from a spherical primordium into a full-sized drum-stick-like young mushroom took several days, 2 to 3 days at warmer days (18–22 °C), while it slowed down up to 6 to 8 days at colder temperature (12–15 °C). Fully grown drum-sticks were up to 10 to 12 cm tall with a cap diameter of about 3 to 5 cm and a white partial veil at the underside of the cap that covered the lamellae (Fig. 1b). During maturation in the following 2 days, the white partial veil perforated with cap extension at the edge of the pileus. The remaining connections ripped apart with further cap opening and gave the stretched pileus a gear-wheel appearance by tooth-like vestiges (Fig. 1c, d). With the ripping, the partial veil stayed first as a well-shaped skirt-like white annulus around the stipe (about 1.0 to 1.5-cm in Ø, with the lower base somewhat swollen) at a distance of about 2 to 2.5 cm beneath the cap, but it degenerated with time over the following days. Opened caps were about 10 to 13 cm in diameter. On the upper surfaces towards the centres of the pilei were small yellowish to light brown scales. With cap opening, the densely arranged masses of initially pinkish thin lamellae (over 60 full length primary lamellae per cap with 5 to 7 secondary lamellae in between) turned quickly dark brown. Within 2 to 3 days, the cap colour turned pale-greyish and, slowly over 10 to 15 days, the open matured mushrooms grew old. The brown thick-walled smooth basidiospores (examples can be seen in Fig. 2m) measured in average 5.05 ± 0.5 × 3.89 ± 0.63 µm (n = 21).

By mushroom morphology and spore sizes, our morphological observations on the mushrooms concur with the descriptions by Breitenbach and Kränzlin (1995) for *A.* *xanthodermus*. However, strong yellow coloration upon injury of stems as typical for the species was first not noted; a faint yellow colour was seen on scratched freshly harvested mushroom stipes in September 2016 and again in July and more intensively in August 2017. The odour of healthy mushrooms of the colony was rather a faint mushroom scent than the typical pungent phenol odour of the species (Gill and Strauch 1984; Petrova et al. 2007) which in contrast was noticed by us for other *A.* *xanthodermus* colonies in the Göttingen-Weende area. Lack of both parameters together initially lead to a misidentification as *Agaricus macrosporus* by its very similar mushroom shapes and sizes (Lakkireddy et al. 2016). The species identity *A.* *xanthodermus* of the mushroom colony underneath the *P. menzii* tree was here confirmed by sequencing ITS DNA which was PCR-amplified from genomic DNA of a stipe of a mushroom harvested on 4th of September 2015. The established sequence (KX098652) was 99 and 100% identical to *A. xanthodermus* sequences AY484689 and DQ182529.1 from GenBank (Geml et al. 2004; Kerrigan et al. 2005).

### Diseased mushrooms of *Agaricus xanthodermus* in nature

On 1st of September 2015, among several normal healthy fruiting bodies, we noticed a crippled young mushroom at the late drum-stick state that had a bended deformed stipe and a split cap (Lakkireddy et al. 2016). A directly neighboured primordial mushroom had dropped and was half-covered by a mycelial white network that extended over the stipe onto the edges of the cap of the other crippled individual (Fig. 1e). Over the next 2 days, the still healthy parts of the cap of the crippled mushroom extended in size to expose the pinkish lamellae while the primordial mushroom degenerated into an amorphous clump under actions of the foreign mycelium (Fig. 1f–i). As seen a day later, cap tissues of the crippled mushroom quickly aged, probably accelerated through the presence of the foreign mycelium. A thick mycelial layer of a fungal infestation was present at the side of the cap that was closer to the ground (Figs. 1j, 2a, b) and as cover over the stipe of the mushroom (Figs. 1k, 2c, d) from which it grew onto the lamellae (Fig. 2d–i). The harvested infected mushroom had an unpleasant smell. Sequencing of ITS DNA (KX098651) PCR-amplified from mushroom tissues again confirmed *A.* *xanthodermus* as the species identity.

Conidiophores with oblong spores were obvious in thick older mycelium grown on the upper side of the cap, on the stipe and the lamellae (Fig. 2b–g). We microscoped mycelial samples from the lamellae and found conidiophores and hyaline dry conidia (Fig. 2j–n) which suggested that the infestation was of the anamorphic genus *Cladobotryum* of the family of *Hypocreaceae* (*Hypocreales*, *Sordariomycetes*) of the *Ascomycota* (Cole and Kendrick 1971). Conidia were one to four-celled (18.0% one-celled, 63.9% two-celled, 9.8% three-celled; 8.2% four-celled; n = 61) with the majority being two-celled as it is typical for e.g. the mycopathogenic type species *C. varium* and *C. mycophilum* (Hughes 1958; Cole and Kendrick 1971; Rogerson and Samuels 1993; Back et al. 2012b; Tamm and Põldmaa 2013). Individual colonies were isolated from mycelium covering the stipe (strains AscoA1 and AscoB1) and from lamellae (AscoC1) of the infested mushroom.

Upon aging, degenerating mushrooms in the meadow were also visibly attacked by similarly sporulating fungi (not further shown). Another mycelial strain (AscoE1) was thus isolated on 11th of September 2015 from a heavily infested rotting stipe of a formerly healthy *A. xanthodermus* mushroom when it was found knocked-down in course of aging on the meadow.

**Fig. 3:**
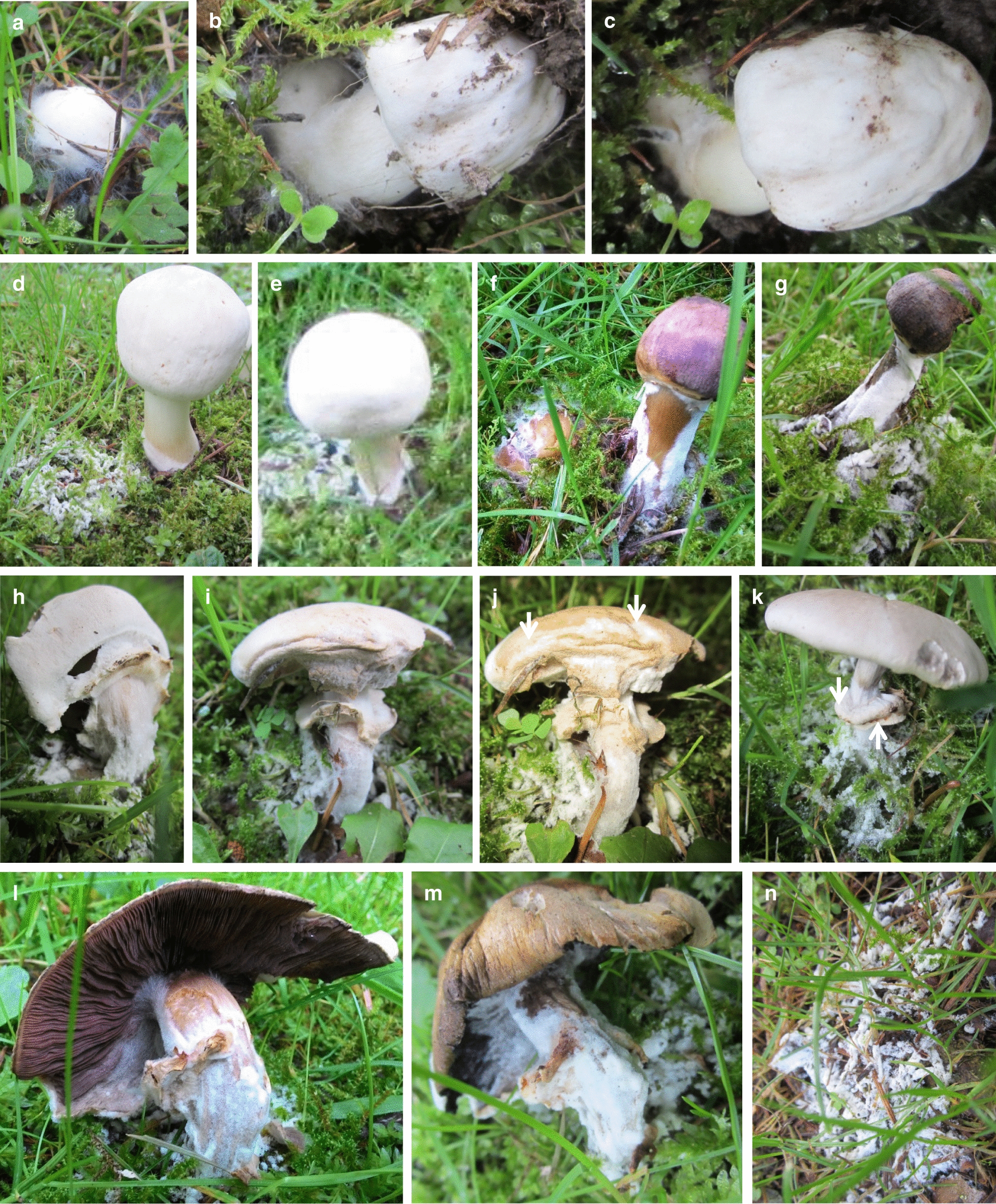
Mycoparasitic mycelium infested *Agaricus xanthodermus* mushrooms at different developmental stages in a larger disease outbreak in the 3rd week of September of 2015. **a**, **b** Foreign mycelium grew from surrounding moss to a primordium and the stipe base of a young mushroom at the stage of stipe elongation and cap growth. **c** 24 h later the stipe base was surrounded by a thick layer of foreign mycelium, the stipe and cap were enlarged but the cap laid down on the floor due to strong bending of the stipe. **d**–**m** Strong white mycelium found at multiple places in the grass and moss served as infection source of *A. xanthodermus*. Bases of elongating stipes of growing drum-stick-like young mushrooms were covered by a layer of foreign mycelium (**d**, **f**) and the same structures 24 h later photographed from different angles (**e**, **g**). While the yet less infected structure with the foreign mycelium confined only to the stipe base was still erect (**e**), the heavily infected structure with foreign mycelium reaching up to the cap already collapsed (**g**). Infested young mushrooms at the start of partial veil rupture (**h**, **i**). 24 h later, the cap of the mushroom shown in **i** coloured brownish and the rupture of the partial veil blocked. Thick white patches of the pathogen were obvious on the cap surface (see arrow; **j**). Also young stages of opened caps (the arrows mark the skirt-like annulus injured by the infestation) were attacked by mycelium growing upwards the stipe (**k**) and eventually also onto the lamellae (**l**, **m**). Rapid decolourization and mushroom collapse within 24 h resulted from strong pathogen infestation (**l**, **m**). Strong white mycelium found at multiple places in the grass and moss (**n**)

Following some heavy rainfall on 14th and 15th of September 2015 with a drop in temperature from the 16–21 °C at previous days, a second flush of *A. xanthodermus* mushrooms was observed in the 3rd week of September 2015, at day temperatures (noon) of 12 to 19 °C. Small spherical primordia were seen first on the 16th of September. Several structures were found 5 days later to be diseased at different developmental stages of mushroom development. Infestations started from white mycelial patches of several cm in diameter that developed first well visible on the 18th of September in the neighbourhoods on moss and decaying grass (Fig. 3), needles and cones (not shown). Sometimes these patches originated clearly from the remains of older mushrooms (Fig. 3f) but there were also multiple patches of fluffy white mycelium that did not obviously connect to a place of former mushroom production (Fig. 3n). Another fungal colony (strain AscoD1) was isolated from a decaying grass and moss sample from such a patch of sporulating white mycelium.

Mushrooms of differential developmental ages became infested by foreign mycelium, even very young primordia (Fig. 3a). Fluffy white mycelium grow onto the lower base of another young mushroom at the beginning of stipe outgrowth and, possibly as a consequence, the stipe of the young mushroom strongly bended with the mushroom cap laying down on the floor (Fig. 3a, b). Erect older drum-stick-like stages with extended stipes were also seen to be confined from the bases of the stipes (Fig. 3d–g). In some instances, heavy infestation lead to reddish-brown to lilac decolourisation of stipes (Fig. 3d, f) and also caps, and to collapse of the young mushrooms (Fig. 3f, g). Also older structures at and after cap opening were attacked by foreign mycelium (Fig. 3h–m). Caps of attacked mushrooms turned brown to blackish-brown and shrivelled, thus quickly grew old (Fig. 3i, j; l, m) and rapidly rotted (not further shown). The reactions on older fruiting bodies appeared to be more aggressive and faster than reactions on younger stages.

### Mycopathogens in culture

All five isolated strains formed conidiogenous mycelium and grew well on MEA at RT (about 22 °C) with increases in colony radii of 3.7 ± 0.2, 3.8 ± 0.3, 3.6 ± 0.1, and 3.6 ± 0.1 mm/day (AscoA1, AscoB1, AscoC1, AscoE1) and of 2.4 ± 0.1 mm/day (AscoD1), respectively. On the nutrient-rich YMG/T, the colonies increased in radius by 4.3 ± 0.1, 4.3 ± 0.1, 4.2 ± 0.2, and 4.4 ± 0.1 mm/day (AscoA1, AscoB1, AscoC1, AscoE1) and 2.0 ± 0.1 mm/day (AscoD1). The odour of the fungi when grown on MEA was pleasant faint sweet aromatic (camphor-like, resembling *Eucalyptus* smell). During growth phases on YMG/T, the odour was also first pleasant faint aromatic to medicine-like but when cultures on YMG/T aged and turned wine-red it became unpleasant sharp. The mycelial scents became stronger on both media with an increase in growth temperature to 28 °C.

**Fig. 4:**
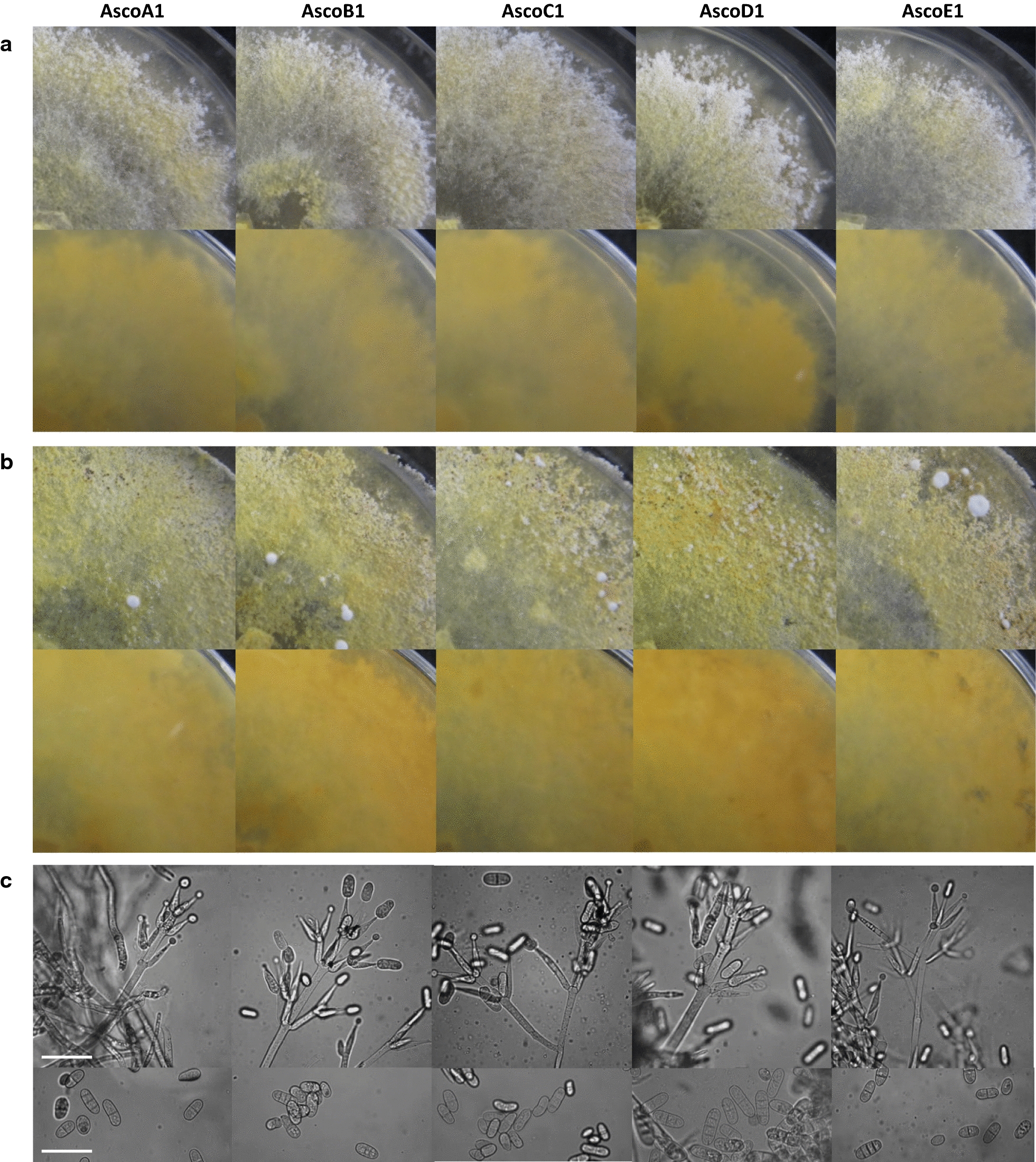
Colony growth of five isolated conidiogenous strains on MEA at RT. **a** After 3–4 days of incubation with white fringed colony borders (upper row: plates from top, lower row: from reverse) and **b** with white pulvinate stroma in fully grown cultures after 25 days of incubation at RT (upper row: plates from top, lower row: from reverse). **c** Morphologies of conidiophores (top) and conidia (bottom) taken from aerial mycelium of growing cultures of isolated *Hypomyces odoratus* strains. Size bars: 50 µm

All five strains grow on MEA at RT as a first slightly pigmented mycelium. Growing colonies on MEA of four of the strains stained first light yellow, while cultures of strain AscoD1 were stronger yellow from the beginning. Comparably little aerial mycelium was produced by all strains resulting in overall flat colony appearances. Growing colonies had small white fringed borders due to the production of multiple conidiophores with white flocks of masses of dry hyaline conidia. Within 2 to 3 days upon production, conidia separated from conidiophores and fell in larger aggregates onto the surface of the yellowish colonies (Fig. 4a). Per fully grown MEA plate, the strains produced between 2 × 10^7^ and 8 × 10^7^ hyaline 0- to 3-septate conidia (Table 1). With time, fully grown colonies turned from slighter yellow to dark yellow (after 4 to 6 days of growth at RT to after about 14 days; when cultured at 28 °C, these processes were 1 to 3 days faster) while strain AscoD1 was still darker pigmented as compared to the others. Cultures appeared to be in a final stage at RT after about 25 days of incubation, for the appearance of mycelium and colony color. The observations on plates cultivated at RT usually lasted up to 36 days as the plates became drier. Mainly the mycelium but also the agar was stained by the strains by yellow pigments. In one experiment when plates were kept for 2 months in very humid conditions, some plates of strains AscoB1 and AscoD1 adopted in the end a mixed yellow-slightly pinkish pigmentation while none of the other strains did change the color. Further in aging MEA cultures after around 3 weeks of incubation, all five strains started to produce hard white patches of dense mycelial pulvinate stroma which increased in numbers with time (between dozens to > 100 per plate). First they were small, less than 0.1 mm in Ø, but with time they could grow to patches of up to 3–4 mm in Ø (Fig. 4b). After around 30 days of cultivation with drying out medium, round dark brown microsclerotia (AscoA1: 0.33 ± 0.08 mm in Ø, n = 16; AscoB1: 0.37 ± 0.08 mm in Ø, n = 13; AscoC1: 0.35 ± 0.08 mm in Ø, n = 14; AscoD1: 0.36 ± 0.07 mm in Ø, n = 17; AscoE1: 0.36 ± 0.07 mm in Ø, n = 15) filled with large round unstained cells formed in aging colonies (not shown). In addition, masses of round chlamydospores (AscoA1: 13.3 ± 1.7 µm in Ø, n = 22; AscoB1: 13.5 ± 1.7 µm in Ø, n = 26; AscoC1: 13.9 ± 1.6 µm in Ø, n = 28; AscoD1: 13.5 ± 2.2 µm in Ø, n = 22; AscoE1: 13.9 ± 1.2 µm in Ø, n = 24) arose in chains from swelling and fragmenting of vegetative hyphal cells (not shown).

**Table 1:** Features of conidiophores of the five isolated strains grown at RT

Cells	Parameter	AscoA1	AscoB1	AscoC1	AscoD1	AscoE1
Conidiophores	Stem					
	1st order whorls	2–5	2–4	2–4	2–4	2–3
	Whorls with branches	0–3	1–3	0–3	1–3	0–3
	Branches per whorl	0–3	1–3	0–3	1–3	0–3
	1st order branches	0–7	1–4	0–3	1–3	1–3
	1st order branch					
	2nd order whorls	0–4	0–3	0–2	0–3	0–3
	Whorls with branches	0–2	0–1	0–1	0–1	0–1
	Branches per whorl	0–1	0–1	0–1	0–1	0–1
	2nd order branches	2	1	1	1	1
	2nd order branch					
	Whorls	2	1	1	1	1
	Branches total	0–4	0–3	0–2	0–3	0–2
	Whorls total	3–7	1–4	1–5	1–4	1–4
	n	15	9	8	14	7
Ampulliform phialides	Per whorl without branches	2–5	2–5	2–5	2–5	2–5
	Per whorl with branches	2–4	2–3	1–3	1–2	1–2
	Length (µm)	35.8 ± 7.7	35.7 ± 7.4	38.1 ± 8.3	31.6 ± 5.8	32.9 ± 5.7
	Apex width (µm)	2.7 ± 0.4	3.3 ± 0.6	3.4 ± 0.5	3.8 ± 0.6	3.1 ± 0.5
	Width broadest point (µm)	8.1 ± 1.1	8.3 ± 1.4	8.1 ± 0.8	8.7 ± 1.2	8.3 ± 1.2
	Base width (µm)	5.2 ± 1.0	4.4 ± 0.7	5.0 ± 1.0	4.6 ± 1.1	4.6 ± 0.9
	n	27	24	16	20	27
Conidia	No septum					
	Length (µm)	15.3 ± 1.2	15.1 ± 1.4	15.5 ± 1.8	15.4 ± 1.6	16.2 ± 1.0
	Width (µm)	10.7 ± 1.3	10.3 ± 1.3	9.6 ± 1.4	10.0 ± 1.0	11.0 ± 1.3
	n	21	13	16	15	13
	% of total	20.0	14.8	14.4	14.9	13.1
	One septum					
	Length (µm)	20.5 ± 3.0	20.7 ± 3.1	21.2 ± 3.3	21.1 ± 4.8	20.8 ± 3.0
	Width (µm)	10.8 ± 1.2	10.3 ± 1.2	10.0 ± 1.3	10.7 ± 1.2	10.4 ± 1.2
	n	71	64	67	64	68
	% of total	67.6	72.7	60.4	63.4	68.7
	Two septa					
	Length (µm)	24.7 ± 3.8	23.4 ± 1.5	24.4 ± 3.7	26.2 ± 4.4	24.3 ± 2.3
	Width (µm)	11.2 ± 1.4	10.5 ± 0.7	10.3 ± 0.9	11.4 ± 1.3	11.2 ± 1.3
	n	12	9	17	16	16
	% of total	11.4	10.2	15.3	15.8	16.2
	Three septa					
	Length (µm)	26.4	25.3 ± 1.4	31.7 ± 3.3	30.9 ± 4.8	26.6 ± 0.1
	Width (µm)	11.9	11.1 ± 0.3	10.5 ± 0.5	12.7 ± 1.3	12.8 ± 1.0
	n	1	2	11	6	2
	% of total	1.0	2.3	9.9	5.9	2.0
	All					
	Length (µm)	20.0 ± 4.0	20.2 ± 3.6	22.0 ± 5.2	21.7 ± 5.8	20.9 ± 3.5
	Width (µm)	10.8 ± 1.2	10.3 ± 1.2	10.0 ± 1.2	10.8 ± 1.3	10.7 ± 1.3
	n	105	88	111	101	99
Conidia per plate	MEA	4.4 ± 1.2 × 10^7^	8.3 ± 4.9 × 10^7^	7.9 ± 2.5 × 10^7^	1.8 ± 1.4 × 10^7^	4.4 ± 1.7 × 10^7^
	YMG/T	1.2 ± 0.6 × 10^8^	1.2 ± 0.2 × 10^8^	9.2 ± 1.3 × 10^7^	2.3 ± 0.1 × 10^7^	9.9 ± 3.9 × 10^7^

Strains were cultivated for 13 days at RT on MEA and conidiophores were taken for microscopy from the outer white growth zone characterized by flocks of conidia. n = number of structures or cells analyzed. For counting spores per plate, five plates per strains and per medium were inoculated and spores were counted after fully growth of plates at RT

**Fig. 5:**
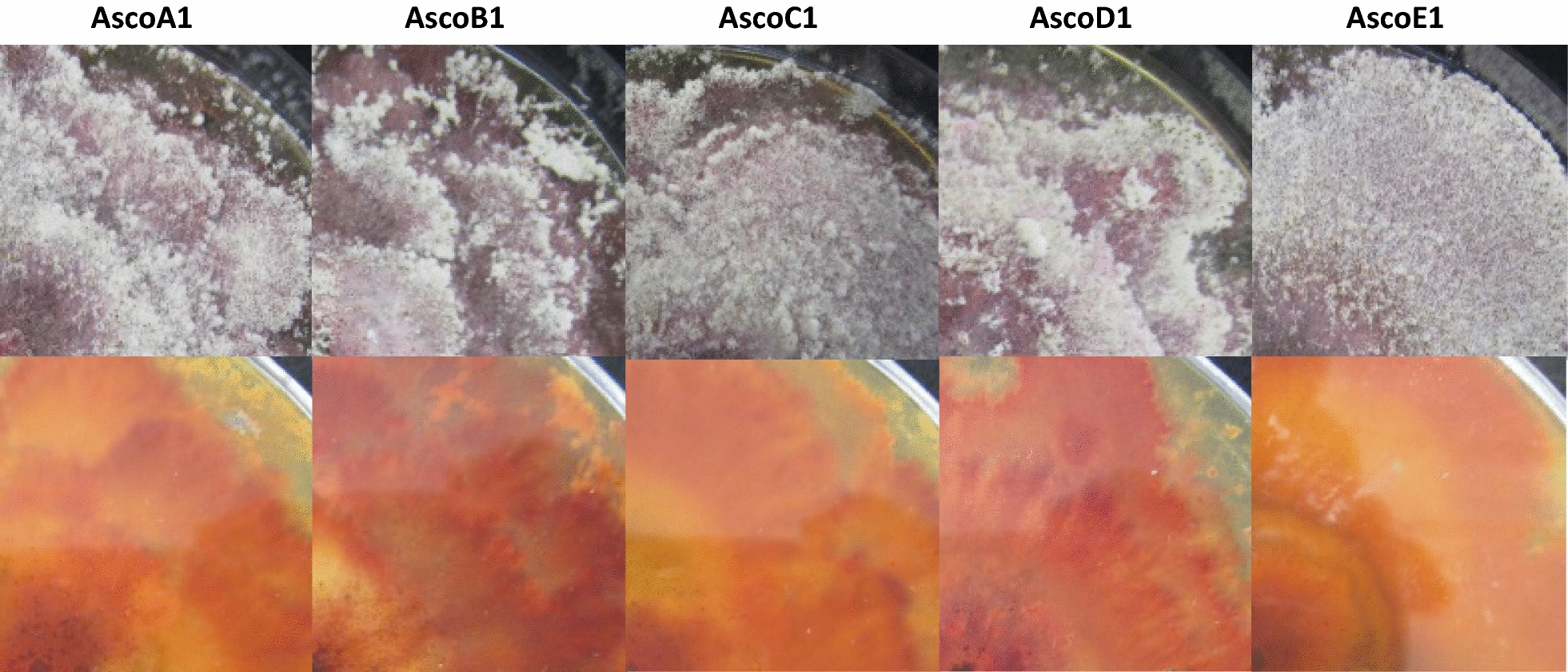
Colony morphology of five isolated conidiogenous strains on YMG/T at RT after 25 days of incubation. Upper row: plates from top, lower row: reverse

Mycelia of all five strains on YMG/T medium were first nearly unpigmented during the fresh growth at RT. The cultures were characterized by loose white fluffy aerial mycelium starting to regularly develop behind the colony growth fronts on the 1-day-old mycelium and to produce conidia over the following days. Spreading in the growing colony outwards from the inoculum, substrate mycelium with the agar began to stain yellowish 1 to 2 days after first aerial mycelium production (at 28 °C 1 or 2 days earlier than at RT), while the yellow colour intensified continuously with further mycelial age in growing. The yellow colour increased in intensity during the further incubation also after plates were fully grown (after 5 and in the case of AscoD1 7 days of incubation) while after about 15 and, in the case of AscoD1, 20 days there was a switch in colour to first light pinkish and later wine-red (Fig. 5). The final cultural stages also appeared to have been reached on YMG/T plates after culturing at RT for about 25 days, followed by only desiccation reactions with continued incubation up to 36 days.

Pink to red medium coloration has been reported before e.g. from older PDA cultures of *Hypomyces*/*Cladobotryum* strains (Back et al. 2010; Carrasco et al. 2016; Muhammad et al. 2019). *Hypomyces*/*Cladobotryum* species are known to produce aurofusarin as pigment (Rogerson and Samuels 1989; Põldmaa 2011; Tamm and Põldmaa 2013; Carrasco et al. 2016) which changes in color from yellow to red depending on the pH (Ashley et al. 1937). We therefore checked the pH in the medium over the time of cultivation. When colonies on YMG/T plates were stained yellowish to dark yellow, the pH in the medium did not much change and was around 5.5 to 6. With onset of pinkish coloration however, the pH increased to values of 6.5 to 7. With increasing colorization when the cultures turned pink to finally wine-red, the pH rendered into the alkaline range to values of around 7 to 7.5 and then to 7.5 to 8. For comparison, the pH in the MEA cultures was in the acidic range with pH 4.5 in slightly yellow cultures and pH 4 and sometimes even pH 3 in dark yellow cultures. In 1-month-old cultures of the stronger yellow cultures of strain AscoD1, the pH raised first slightly to pH 5. In 2-month-old plates of strain AscoD1 and also of strain AscoB1, within a few days under color changes to mixed yellow-pinkish and then yellowish-pink, the pH raised further to 6 to 6.5 and then pH 7. Cultures of strains AscoE1, AscoA1, and finally AscoC1 also increased in pH to 6 but two to several days later, along with color changes into yellow-pinkish.

The pigments in YMG/T cultures stained majorly the submerged mycelial agar layer and to less part the agar beneath. Notably, the colony surfaces remained white in appearance due to the considerable amounts of whitish aerial mycelium with huge amounts of hyaline conidia produced (for spore numbers per fully grown plates see Table 1). The dry conidia assembled into larger flocks on the tips of the conidiophores. During colony growth, thick aerial mycelium arose as high as up to the lids of the Petri dishes, transferring large parts of the clumps of spores onto the plastic surface (not shown). This thick aerial mycelium was longer lasting. After mycelial growth on a plate was completed, and after the change in colour of the substrate mycelium with agar from yellow to wine-red and along with the evaporation of any humidity from the lids of the Petri dishes (after about 25 days of incubation), the aerial mycelium in undisturbed plates collapsed slowly throughout the colony. With opening the lid however, the aerial mycelium collapsed immediately. Eventually, the aggregated conidial clusters fall down from aerial mycelium and lids of Petri dishes in irregular patterns onto the surfaces of the colonies. Spore aggregates collected from agar and from lids of Petri dishes needed harsh forces to separate them into individual cells for counting (Table 1). Furthermore, all strains produced on YMG/T on the surfaces of aging cultures (after about 30 days of cultivation, mainly in the outer regions of colonies) also masses of dark brown microsclerotia which were much more in numbers but of similar sizes than those on MEA (AscoA1: 0.40 ± 0.08 mm in Ø, n = 15; AscoB1: 0.39 ± 0.05 mm in Ø, n = 20; AscoC1: 0.37 ± 0.05 mm in Ø, n = 16; AscoD1: 0.35 ± 0.03 mm in Ø, n = 20; AscoE1: 0.32 ± 0.03 mm in Ø, n = 22). Aging cultures on YMG/T did not produce white stromas but they gave rise to some chlamydospores resulting in chains from swellings and fragmenting of hyphal cells (AscoA1: 13.3 ± 1.4 µm in Ø, n = 22; AscoB1: 13.5 ± 1.5 µm in Ø, n = 21; AscoC1: 13.2 ± 1.8 µm in Ø, n = 20; AscoD1: 12.9 ± 1.6 µm in Ø, n = 24; AscoE1: 13.4 ± 1.7 µm in Ø, n = 23).

### Species identification

Conidiophores with conidia were analyzed in more detail from the strains grown on MEA (Table 1). Conidiophores with conidia on mycelia of all five strains were verticillate as typical for the *Hypomyces*/*Cladobotryum* genus. Conidiophores were separated over their length into several cells. They had stems with 2 to 5 whorls with up to 5 phialides each and they were usually irregularly branched, with 1st order sidebranches arising in numbers between 1 and 3 among some phialides at the lower whorls of the stem and with some 2nd order sidebranches arising at the lower whorls of 1st order sidebranches (Fig. 4c; Table 1). The up to 5 phialides per whorl were successively produced (Fig. 4c and see also Fig. 2j–n) and grew into lengths of > 30 µm (Table 1). The ampulliform phialides tapered from broader regions (width > 8 µm) shortly above their bases (width ca. 5 µm) to slim blunt apexes of widths of around 3 µm. Conidiospores were produced at the simple tips of the ampulliform phialides in monoblastic mode (Fig. 4). First, the young blastospores were equally swelling but with increase in size, they often buckled with further growth to the lateral side (Fig. 4c and see also Fig. 2j–n). Released conidia were hyaline, oblong in shape with rounded edges, had sometimes visibly a hilum at the basal ends and different numbers of septa (Fig. 4c). The majority of conidia of all strains (60–72%) were two-celled. However, strain AscoA1 had more non-septated spores (20%, comparably to the mycelium grown on the infected cap from which AscoA1 was isolated, please see above) than the other strains. Strains AscoC1, AscoD1 and AscoE1 had higher numbers of spores with two or also three septa (in sum 18.2 to 25.2%; Table 1). Between the strains, there were some measured minor size variations of the spores (Table 1). Spore lengths ranged from 13.2 to 33.0 µm (AscoA1), 13.4 to 28.8 µm (AscoB1), 11.7 to 36.5 µm (AscoC1) and 13.6 to 37.4 µm (AscoD1), 13.8 to 28.2 µm (AscoE1). In tendency, spore lengths and widths increased with numbers of septa (Table 1). The strains AscoC1 and AscoD1 with higher percentages of both 3- and 4-celled spores had thus a bit more of the longer spores as compared to the other three strains.

The general morphological parameters of conidiophores and conidia of the five strains matched descriptions of *H. odoratus*/*C. mycophilum* in the literature (Arnold 1963; Gams and Hoozemans 1970; Cole and Kendrick 1971; Gray and Morgan-Jones 1980; Back et al. 2012b; Tamm and Põldmaa 2013; Gea et al. 2014). The occurrence of microsclerotia and presence of round chlamydospores in the aged mycelium and yellow to red stained colonies with a strong smell on nutrient-rich YMG/T medium also concur with descriptions of *H. odoratus*/*C. mycophilum* (Helfer 1991; McKay et al. 1999; Grogan 2006; Gea et al. 2014; Carrasco et al. 2017). In other instances reported in the literature, no peculiar stronger smell was noted by isolates of *H. odoratus* (McKay et al. 1999; Gea et al. 2019), similar as in this study when growing the five strains on MEA plates.

We amplified and sequenced the 530 bp long ITS rDNA regions of all five isolates (KX098646-KX098650). The sequences of the strains are identical to each other and 99–100% identical to the ITS sequences of *H. odoratus* (FN859435; Põldmaa 2011) and *C.* *mycophilum* strains (JF693809, JF505112, AB527074, JQ004737, Y17094, Y17095, KP267826) shown to infect mushrooms in culture (McKay et al. 1999; Back et al. 2010; Kim et al. 2012; Carrasco et al. 2016; Gea et al. 2016). In contrast, they were only 98% identical to *H.* *rosellus* (FN859440, FN859442; Põldmaa 2011) and *C. dendroides* ITS sequences (Y17090, Y17092; McKay et al. 1999). Three subgroups of ITS fragments of *H. odoratus*/*C.* *mycophilum* strains are distinguished (McKay et al. 1999; Tamm and Põldmaa 2013; Gea et al. 2016) by a 1 base pair difference in the ITS1 sequence (base A at position 80 in subgroup 1/2 versus G in subgroup 3) and 1 or 2 base pair differences in the ITS2 region (base T at position 390 in subgroup 1/2 versus C in subgroup 3; base C at position 507 in subgroup 1 versus T in subgroups 2/3). Our sequences fall into *H. odoratus*/*C. mycophilum* subgroup 3 together with strains from Ireland, Estonia, Russia and the USA (McKay et al. 1999; Tamm and Põldmaa 2013).

### Fruiting body infection tests

**Fig. 6:**
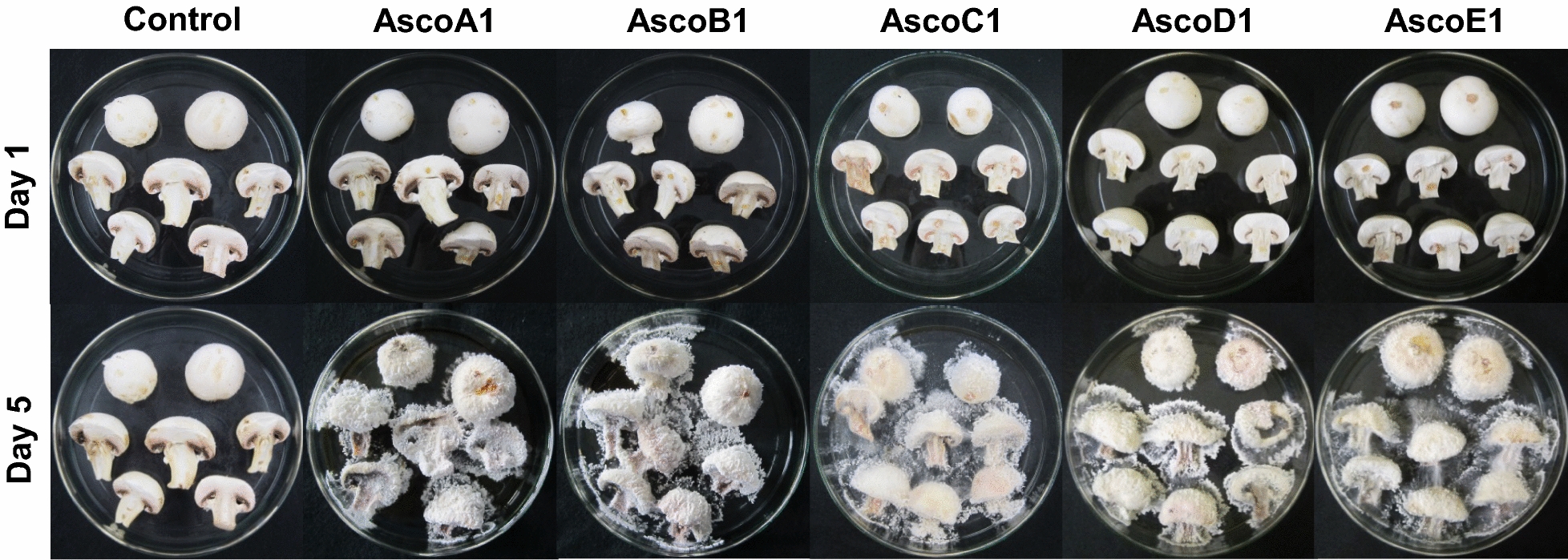
Infection test with commercial *A. bisporus* fruiting bodies. Top row: day of inoculation, bottom: mushrooms after 5 days of cultivation at RT

All five isolated *H. odoratus* strains were tested on complete or halved commercial mushrooms of *A. bisporus*. All five strains regularly infected all commercial *A. bisporus* mushrooms, regardless of whether the inoculum was placed onto a non-injured stipe or cap or onto cuts of stipes and caps of sliced mushrooms (Fig. 6). After placing fresh mycelial MEA agar blocks of the ascomycetes onto a stipe or a cap region of *A.* *bisporus*, the hyphae started to grow (1st day). When intact pilei were inoculated, the surrounding *A. bisporus* cap region in consequence caved in with a growing pathogen, resulting in a visible dent with the inoculum in the center (2nd day). Later on, regardless of place of inoculation, the hyphae spread over all the mushrooms (3rd day) and produced huge white-coloured masses of conidia. During this time, the pathogens were very aggressive and appeared to absorb nutrients from the mushrooms, while the mushrooms reduced in sizes and weights, changed in color from white to light brownish, and became watery-rotten (5th to 6th day). All infectious strains, AscoA1 to AscoE1 gave rise to black microsclerotia on the overgrown surface of the *A. bisporus* samples (not further shown).

**Fig. 7:**
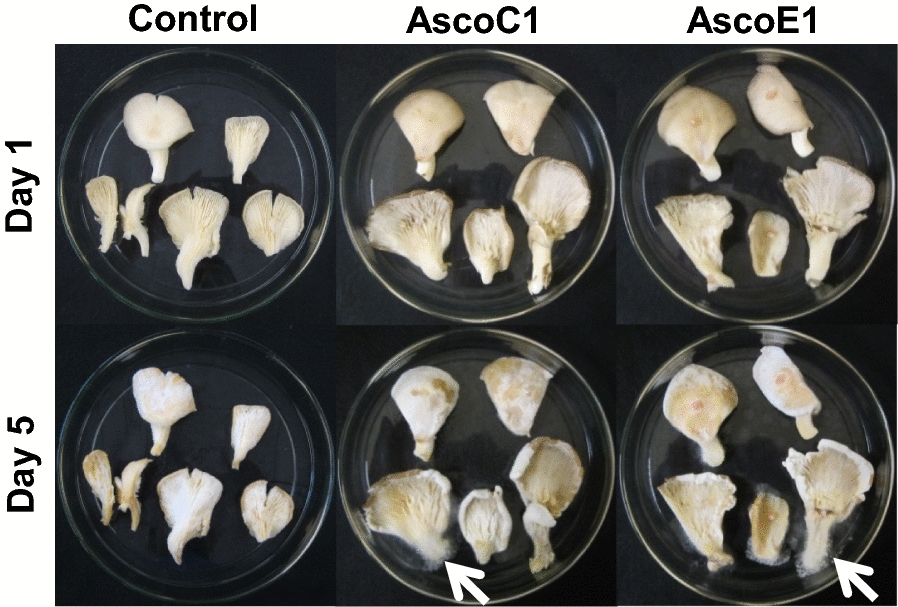
Infection test with commercial *P. ostreatus* fruiting bodies. Top: day of inoculation, bottom: mushrooms after 5 days of cultivation at RT. Arrows mark infested stipe regions. Other mycelial outgrowth noticed came from *P. ostreatus*

In contrast to fruiting bodies of *A. bisporus*, strains AscoA1, AscoB1 and AscoD1 did not grow much on commercial *P. ostreatus* fruiting bodies, neither when inoculated on the cap surface nor on the lamellae nor on the stipes. *P. ostreatus* thus showed resistance to the ascomycetes. At most, the *H. odoratus* hyphae spread from the inocula only over very small areas of the mushrooms without obvious symptoms of disease but not over the complete mushrooms. Importantly, when placing a mycelial agar block at the centre of mushroom caps, the *H. odoratus* mycelia started to grow out more likely on the side of the agar blocks towards the stipe region than upwards of the cap region. Only strains AscoC1 and AscoE1 showed in exceptional cases some infection by mycelial growth on the base of *P. ostreatus* stipes (noticed on each 2 of 25 in total tested fruiting bodies; Fig. 7). Still, also in these rare cases the aggressiveness towards *P. ostreatus* was comparatively low with few amounts of conidia formed by the growing mycelium. As a further interesting observation, *P. ostreatus* tissue growth (growing hyphae had clamps) occurred at the stipe margins, cap margins and the lamellae of inoculated mushrooms and also of uninfected controls. Such growth probably strengthened the mushrooms and helped in resistance against the ascomycetes.

**Fig. 8:**
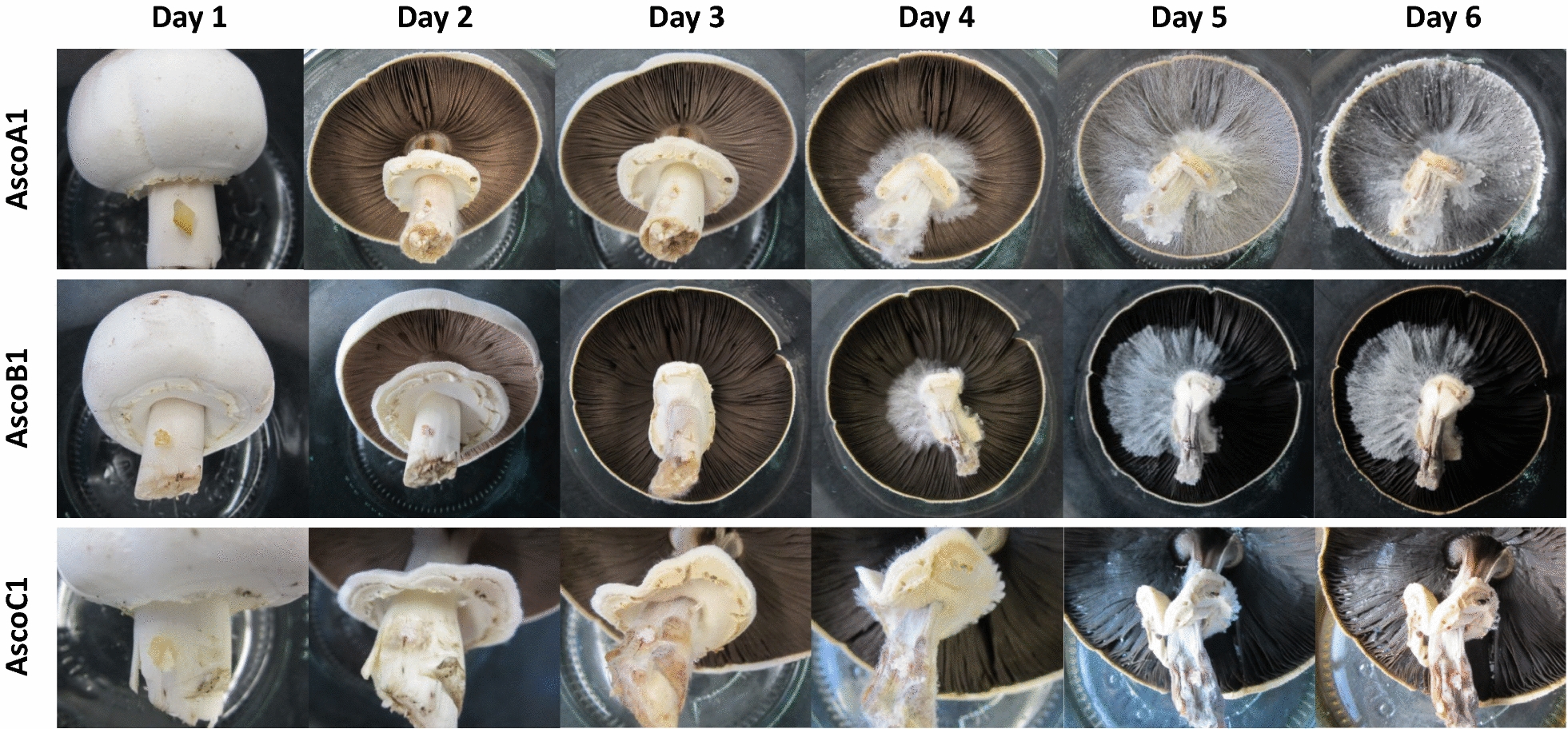
Young *A. xanthodermus* fruiting bodies inoculated with *H. odoratus* strains. Upon stipe inoculation of mushrooms kept within sterile glass jars, the progress in mushroom development and pathogen infestation at RT was photographed once per day

We also tested in similar manner *A. xanthodermus* mushrooms collected from the wild (KX098653) with *H. odoratus* strains AscoA1, AscoB1 and AscoC1. Young mushrooms with still closed caps were infected by agar pieces with the ascomycetes positioned either at the stipes or the caps. Within 6 days at RT, mycelium from the inocula of the stipes grow onto the darkened gills of the matured mushrooms while stipes with the annuli degenerated whereas the caps still remained in good shape (Fig. 8). In contrast upon inoculation of pilei, tissues overgrown by the pathogens shrivelled under appearance of liquid yellow–brown droplets on the cap surface and the caps degenerated quickly (not shown). Similar observations were made, when *A. arvensis* mushrooms from the wild (KX098654) were inoculated with strain AscoC1 (not further shown).

### Infection tests of growing mycelial cultures

**Fig. 9:**
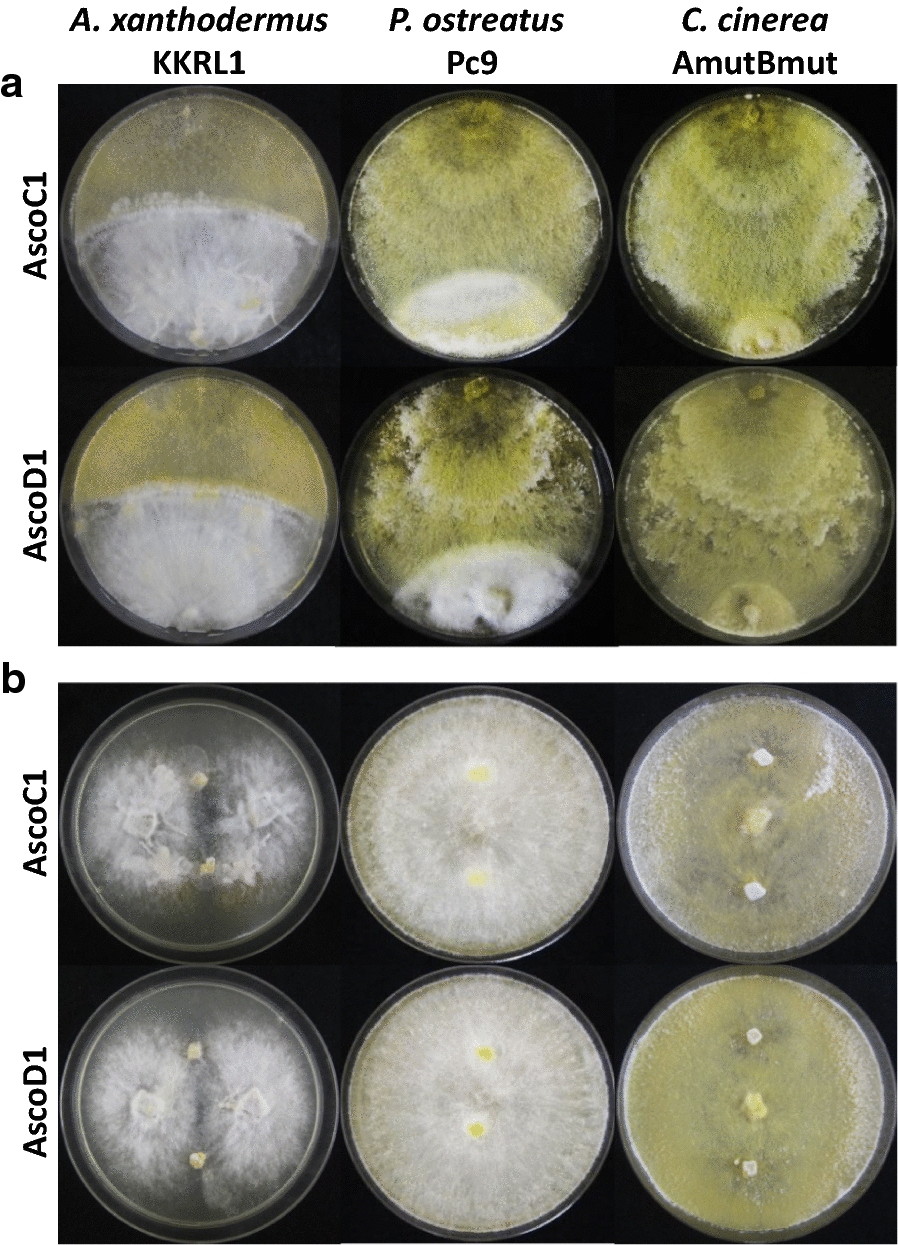
Mycelial growth confrontation tests (top rows) and grown mycelium challenge tests (bottom rows) of *A. xanthodermus* KKRL1, *P. ostreatus* Pc9 and *C.* *cinerea* AmutBmut by *H. odoratus* isolates on MEA, photographed after 20 days of incubation at RT after inoculation of the pathogen

The infection potential of the five mycopathogenic strains was further tested against mycelial cultures of *A. xanthodermus*, *P. ostreatus* and *C. cinerea*, respectively. For mycelial confrontation tests, we inoculated mycelial agar plugs from a test fungus and a respective mycopathogen onto MEA plates at opposite edges of Petri dishes (Fig. 9a).

The dikaryotic *A. xanthodermus* isolate KKLR1 grew slowly on MEA at RT (about 1.4 ± 0.1 mm/day) with a flat cottony-dense white-colored mycelium growing in a loosely organized dense fan-strand pattern. Therefore for the mycelial confrontation tests, the species was inoculated at the edges of Petri plates and cultivated 15 days prior to the inoculation of the mycopathogens at the edges of the opposite side of the plates (see examples of AscoC1 and AscoD1 confrontation tests in Fig. 9a). Once the mycelial growth fronts of the two species reached each other in further incubation (after about 12 to 18 days of incubation), both *H.* *odoratus* as *A. xanthodermus* colonies were stopped in further growth at the confrontation zones with some combat reactions at the colony borders. The flat dense white mycelium of *A.* *xanthodermus* showed some resistance against overgrowth by the mycopathogens. However, bunches of sporulating *H.* *odoratus* aerial hyphae were observed to grow over the *A.* *xanthodermus* colonies. The mycopathogens produced huge amounts of dry aerial conidia which landed in clumps also on *A.* *xanthodermus* mycelium but without recognisable germination. Unevenly distributed, ribbon or thread-like mycelial aggregates appeared as deformations in the *A.* *xanthodermus* colonies (Fig. 9a) and, with longer incubation time, on their surfaces also faint zones of yellowish-stained aerial *H.* *odoratus* mycelium overlaying the basidiomycete. Further in older cultures, *A. xanthodermus* appeared to produce a new thicker white aerial mycelium at the colony borderlines which grow a few mm to over 1 cm into the zones of the opponent colonies and covered the edges of the *H.* *odoratus* colonies (Fig. 9a). *A. xanthodermus* in single culture on MEA plates rarely produced clamp cells at its hyphae. However, some clamp cells were also observed on growth fronts of the new white mycelium overgrowing the *H.* *odoratus* mycelium, supporting that the basidiomycete survived and revived in the dual cultures.

Observation with *A.* *xanthodermus* strain KKRL1 on YMG/T plates were in parts similar. *A.* *xanthodermus* colonies were for 4 weeks pregrown into colonies of ca. 3 to 3.5 cm in diameter prior to inoculation of the mycopathogens. The overgrowth of *A.* *xanthodermus* colonies by aerial mycelium of *H.* *odoratus* strains was then stronger with fast growing thick bunches of conidiogenous hyphae attracted to and overlaying densely the *A.* *xanthodermus* mycelium. Masses of white clumps of conidia were produced on the plate and fell over the covered *A.* *xanthodermus* mycelium. The areas on the plates with the grown *H.* *odoratum* colonies turned lilac-red unlike the unstained agar underneath the covered *A.* *xanthodermus* mycelium. On the reverse of the cultures, dense assemblies of many submerged brown microsclerotia filled with large round cells appeared underneath the overgrown *A.* *xanthodermus* colonies and often also underneath in the agar zone around the *H. odoratum* inocula. Microsclerotia were also observed above the overgrown *A.* *xanthodermus* mycelium. Mycelial samples from *A.* *xanthodermus* colonies overgrown by *H. odoratus* strains revealed under the microscope single-celled chlamydospores and many conidia of the ascomycete. No new outgrowth of mycelium was observed on plates from the *A.* *xanthodermus* colonies which might not have been strong enough for such activity if still alive.

In mycelial confrontation experiments with *P. ostreatus* monokaryon Pc9 on MEA, the mycopathogens grow also faster than strain Pc9 and the yellow stained colonies produced huge amounts of conidia (Fig. 9a). Where the growing species met, combat reactions resulted, leading to a margin of denser white mycelium formed by the Pc9 strain as delineation from the yellowish mycopathogens. However, very long conidiogenous hyphae of the mycopathogens loosely overgrow the *P. ostreatus* colonies and conidia were produced in small white flocks especially at the plastic edges of Petri dishes above the *P. ostreatus* colonies. With time, some mycelial patches above the Pc9 mycelium stained yellowish. In mycelial samples from the *P.* *ostreatus* colonies under the microscope, no or only few mostly two-celled conidia were detected. From the reverse of plates, sometimes thinner necrotic areas became visible in the unstained *P.* *ostreatus* colonies of older plates (1 month) while in other areas and cases the mycelium grew denser. In mycelial confrontation tests on YMG/T medium with much more production of aerial *H. odoratus* mycelium, reactions were much stronger. Conidiogenous hyphae were strongly attracted in growth to the Pc9 colonies for covering the colonies, and masses of conidia in many very large aggregates were produced above the *P.* *ostreatus* mycelium. On the reverse side of cultures, production of some brown microsclerotia were observed underneath in the unstained *P. ostreatus* colonies, while all other parts of the medium covered with mycelium of *H. odoratum* strains were stained pink to lilac-red.

The third species tested, *C. cinerea* homokaryon AmutBmut, in contrast was not able on MEA to defeat any of the five mycopathogens in combat reactions. *C. cinerea* was easily overgrown by all five *H.* *odoratum* strains when the growing colonies were confronted with each other. *H.* *odoratum* strains formed regular yellow colonies with also regular conidia production over the whole plates including the growth zones of *C. cinerea* (Fig. 9a). Because *C.* *cinerea* AmutBmut is a self-compatible homokaryon by mutations in its mating type loci, it forms clamp cells at its hyphal septa (Swamy et al. 1984). In mycelial samples of overgrown *C. cinerea* colonies underneath the microscope, clamp cells at hyphal septa were only exceptionally seen, suggesting that at least parts of the existing mycelium probably came from the mycopathogens. White pulvinate stroma developed on top of the *C.* *cinerea* colonies in 1 month old MEA plates. In confrontation tests on YMG/T plates, *C.* *cinerea* colonies of equal growth age than the mycopathogens were also quickly overgrown by the *H.* *odoratus* strains through outgrowth of dense fast growing hyphal fans of *H. odoratus* mycelium being attracted to the smaller *C. cinerea* colonies. Masses of conidia were produced on top of the *C. cinerea* colonies while the edges of the colonies were less sharp and, as seen on the reverse of the plates, the pink *H. odoratus* staining diffused into the borders of the *C. cinerea* areas. In confrontation tests with larger pregrown *C. cinerea* colonies (inoculated at edges of plates and incubated 4 days at 37 °C prior to inoculation of *H.* *odoratus* strains and transfer to RT), defense was stronger with sharper colony borders against the mycopathogens. However, the surfaces of the *C. cinerea* mycelia were also quickly covered by fast growing conidiophores and masses of conidia.

### Infection tests of grown mycelial cultures

In other experimental series to challenge a grown test fungus, mycelial agar plugs of mycopathogens were placed at 2 cm distance from inocula on the top of the completely grown basidiomycete mycelium (Fig. 9b). In the grown mycelium challenge tests with already established mycelium (grown with two inocula per MEA plate for 20 days at RT), the mycelium of *A. xanthodermus* could well resist the five mycopathogens. In the basidiomycete colonies, some white thread- or ribbon-like or in addition also globular compact mycelial aggregates were detected as reactions (Fig. 9b), similar as before in the confrontations tests on the same medium. Clamp cells were detected in the mycelium. When using slowly growing *A.* *xanthodermus* colonies on YMG/T medium for surface inoculation with the *H. odoratus* strains, outgrowth of the mycopathogenic strains on the basidiomycetous colonies was impeded unlike on free agar surfaces.

In mycelial challenge tests, we also noticed on both media little or no outgrowth of mycopathogens when inoculated on the top of established Pc9 mycelium (Fig. 9b). Only sometimes in closer vicinity of the inocula of *H. odoratus* strains, zones of some denser mycelium or some minor necrotic reaction were observed. Like the fruiting bodies of the species, also the vegetative mycelium of *P.* *ostreatus* exerts thus some but not full resistance against the mycopathogens.

No much outgrowth of the mycopathogens was then observed when inoculated on YMG/T plates that were fully grown with dense *C. cinerea* mycelium (inoculated in the middle of plates and grown for 6 days at 37 °C). When inoculated on top of established but less dense *C.* *cinerea* mycelium on fully grown MEA plates, the *H.* *odoratus* strains however could easily overgrow the *C. cinerea* mycelium and surfaces of colonies stained yellowish by the presence of the mycopathogen (Fig. 9b). Necrotic areas became visible in the *C. cinerea* colonies underneath by thinned mycelium around the inocula of the mycopathogens in mycelial challenging tests on MEA (Fig. 9b).

## Discussion

In this study, we report observations on mycoparasitic infections of *A. xanthodermus* mushrooms in nature. We have observed unimpeded developing mushrooms in years 2012 to 2017, variably in the months June, August, September and, in 2015, also in November, usually after comfortably warm weather conditions. Induction of fruiting body development of *A. xanthodermus* seems to need sufficient previous rainfall possibly to both, moisture the ground and create higher humidity in the air. Consequential to the rainfall, a drop in air temperature likely will also be favourable for induction of fruiting. Fruiting body development proceeds from ball-like primordia over drum-stick-shaped, still closed young mushrooms to mature mushrooms with open umbrellas and first pinkish and then brown lamella (Fig. 1). The speed of development from primordia to fruiting body maturation seems to depend also on the temperature and took in our observations between 10–13 and 6–8 days at colder and warmer temperature (around 12–15 °C and 18–22 °C), respectively. Mature fruiting bodies can last further 10 to 15 days.

### Infections of *A. xanthodermus* fruiting bodies by *H. odoratus* cobweb in nature

Interestingly, in a first flush of mushrooms in early September 2015, one split fruiting body was visibly affected by a fungal infestation (Figs. 1e–l, 2). While we do not know whether this single mushroom was injured prior to infestation or whether infestation resulted in the injury, our observations from infections in the subsequent flush of mushrooms suggest that injury is not a premise of infection of the species in nature. Moreover, we observed that all stages of fruiting body development were susceptible for the mycopathogen (Fig. 3). The infections on *A. xanthodermus* were identified by morphological means (conidiophores and conidia) and ITS sequencing as *H. odoratus* (anamorph *C.* *mycophilum*). This fungus is one of a group of closely related species which can cause cobweb disease of cultivated mushrooms such as the edible species *A. bisporus*, *P. eryngii* and *P. ostreatus* (see e.g. Back et al. 2012b; Tamm and Põldmaa 2013; Gea et al. 2014, 2016, 2019; Carrasco et al. 2016; Chakwiya et al. 2019). The species proliferates also on mushroom substrates (Grogan 2006; Carrasco et al. 2016; Gea et al. 2016, 2019) and has also been encountered on the polypores *Ganoderma lucidum* (Zuo et al. 2016) and *Polyporus* sp. in culture (Rogerson and Samuels 1994). In commercial button mushroom cultures, any mushrooms encountered will be engulfed by the mycopathogen with radial outgrowth of mycelium on the substrate (Grogan 2006; Muhammad et al. 2019). We have observed similar events in nature with the mycopathogens growing from the surroundings (decayed fungal material, decaying grass/moss, soil) onto the stipes of nearby developing *A. xanthodermus* structures (Fig. 3).

### Spread of *H. odoratus* cobweb clones in nature

Cobweb disease can be spread by airborne conidia. In mushroom-growing rooms, the large conidia are released from spore clusters on the colonies into the air by physical disturbances such as by watering. When subsequently landing and germinating on mushrooms, disease symptoms can be incurred (Dar 1997; Adie et al. 2006; Grogan 2006). Following initially a single infested mushroom (Figs. 1e–l, 2), we observed a larger outbreak of disease in nature after heavy rainfalls in the 3rd week of September 2015 in the 2nd flush of mushrooms of *A. xanthodermus* (Fig. 3). It is thus possible that the rainfall helped to distribute conidia from the place of the previous single mushroom infestation over the larger area, in addition to the general promotion of host and pathogen growth by providing good levels of humidity through rainfall to both. *H. odoratus* conidia do not survive long under dry conditions (Lane et al. 1991) and high humidity is needed for dispersal and germination (Carrasco et al. 2016, 2017). Attack of *A.* *bisporus* by *H. odoratus* in commercial cultures can happen at any stage in the fruiting body development (Carrasco et al. 2017; Chakwiya et al. 2019) while infections tend to become more severe on the crop in later flushes at longer time of cultivation and during autumn and winter cycles with increasing conidia numbers (Carrasco et al. 2016, 2017). Our observations on *A.* *xanthodermus* in nature resemble the reports on disease on *A. bisporus* in commercial mushroom production.

While *H. odoratum* is shown to produce perithecia with ascospores in culture (Arnold 1963; Põldmaa 2011; Tamm and Põldmaa 2013), it is not known to do so in nature. Clonality is expected to occur in nature of asexually reproducing *Hypomyces* species in course of spreading of conidia as a major mode of reproductive distribution (McKay et al. 1999; Grogan and Gaze 2000; Valdez and Douhan 2012; Tamm and Põldmaa 2013; Carrasco et al. 2017; Chakwiya et al. 2019). We isolated five *H. odoratum* strains from a close neighbourhood, three of a same infested fruiting body (AscoA1, AscoB1, AscoC1) but of different mushroom organs (from cap and stipe, respectively). Another isolate (AscoE1) came from a decaying stipe of a later infested mushroom. Their properties were very similar, in measurements only distinct in some minor details. The 5th strain (AscoD1) isolated from grass/moss was more different from the other four such as by slower growth speed, a stronger yellow colony colour and by lower spore production. This might suggest that they are not (all) clonal in relation to each other. Larger population field studies on *H. odoratus* in nature are currently missing in order to know how much genetic diversity exists in natural populations and whether sexual reproduction and recombination occurs in nature. Nearly identical clones have been isolated from commercial *A. bisporus* cultivations in different European countries and other continents. Using worldwide the same *A. bisporus *production strain and spawn and casing soils from same sources, this could however relate to human activities in mushroom cultivations if hygienic conditions were not strictly kept. Further alternative sources of primary infections in commercial mushroom cultures were by human movements and other material transport (Carrasco et al. 2017; Chakwiya et al. 2019). In contrast, clones from mushroom farms have in some instances been interlinked to local populations in nature (Tamm and Põldmaa 2013). There is thus also a possible danger for introduction of the pathogens into mushroom farms newly from the nature.

### Outbreaks and host range of *H. odoratum* cobweb

Most of the present knowledge on the species *H. odoratus/C. mycophilum* comes from cobweb outbreaks experienced in newer time in commercial mushroom cultivations (see “Introduction”; Grogan 2006; Tamm and Põldmaa 2013). In essence, cobweb disease in mushroom cultures is caused by different species and up to recently, there was much confusion on species identities. *H. odoratus/C. mycophilum* was often mistaken by *H.* *rosellus*, a related species with similar disease symptoms. *H. rosellus* has however distinct conidiophores with a rachis at the apex of phialides, produces only two-celled conidia, has a more confined host-range and appears to be less often prevalent in the wild. In addition, the two species differ in their ITS sequences allowing to distinguish the two species further by molecular data why several misidentified strains were later reassigned to *H. odoratus/C.* *mycophilum* (McKay et al. 1999; Tamm and Põldmaa 2013). Our morphological and molecular data define the five strains isolated in this study from the wild clearly as *H.* *odoratus*.

*H. odoratus* has a very broad host range on mushrooms growing in nature in temperate regions (Tamm and Põldmaa 2013). Incidences of infections on agaric fruiting bodies in the wild have sporadically been recorded before for *A. xanthodermus*, *Armillaria mellea*, *Calocybe gambosa*, *Cortinarius collinitus* var. *mucosis*, *Enteloma clypeatum*, *Hebeloma* sp., *Hygrophorus camarophyllus*, *Inocybe* sp., *Lycoperdon pyriforme*, *Megacollybia platyphylla*, *Mycena galericulata*, *Oudemannsiella platyphylla*, *Pholiota* sp., *Pseudoclitocybe cyathiformis*, and *Tricholoma terreum* as well as occurrence on soil, leaf litter and rotting wood (Arnold 1963; Gams and Hoozemans 1970; Helfer 1991; Rogerson and Samuels 1994; Tamm and Põldmaa 2013). *H. odoratus* is considered to be agaricicolous (Rogerson and Samuels 1994) whereas other *Hypomyces*/*Cladobotryum* species are specified as boleticolous and polyporicolous (Rogerson and Samuels 1989, 1993; Tamm and Põldmaa 2013). However, *Coniophora* sp., *Suillus aeruginascens* and *Suillus bovinus* from the *Boletales* (Arnold 1963; Rogerson and Samuels 1994; Tamm and Põldmaa 2013), *Albatrellus* sp., *Lactarius mitissimus*, *Lactarius deliciosus*, *Lactarius quietus*, *Lactarius* cf. *vellereus*, *Russula virescens*, *Russula* sp., and *Stereum sanguinolentum* from the *Russuales* (Arnold 1963; Gams and Hoozemans 1970; Helfer 1991; Tamm and Põldmaa 2013), and *Trametes versicolor* from the *Polyporales* (Gray and Morgan-Jones 1980) are further named as potential hosts for *H.* *odoratus* in nature, as well as *Cantharellus cibarius* from the *Cantharellales*, *Gloeophyllum sepiarium* from the *Gloeophyllales*, and *Clavariadelphus truncatus* from the *Gomphales* (Helfer 1991). Newer observations on the species in nature with molecular identification would be helpful to unambiguously confirm these claims.

Other than the many incidences in commercial mushroom cultivations and the mostly older reports on occasional fungal collections in the wild, little is so far known on the ecology of necrotrophic *Hypomyces* species such as *H. odoratus* in nature. Our observations in nature and the infection tests in the laboratory confirm *A. xanthodermus* fruiting bodies to be susceptible to *H. odoratus*. The host range of the five isolated strains does not restrict to *A.* *xanthodermus* but include further *Agaricus* species. The strains grew on and quickly decayed commercial fruiting bodies of *A. bisporus*, in accordance with the various reports in the literature on occurrence of the species on the white button mushroom in cultivation (see “Introduction”; Grogan 2006; Tamm and Põldmaa 2013). The host range of the five strains extends also onto mushrooms of an *Agaricus* sp. from the section *Arvenses* but not particularly to fruiting bodies of *P. ostreatus*. Resistance against *H. odoratus* has been reported from infection tests for *Hypsizygus marmoreus* fruiting bodies (Back et al. 2012a, b, 2015) whereas *F. velutipes* (Back et al. 2012b), *G. lucidum* (Zuo et al. 2016), *P. eryngii* (Back et al. 2012b; Kim et al. 2014; Gea et al. 2011, 2014, 2016, 2017) and *P. ostreatus* (Pérez-Silva and Guevara 1999; Gea et al. 2019) were found to be (partially) susceptible. However, the place of inoculation can play a role. Upper parts of intact caps of *P. eryngii* were thus relatively resistant against *H. odoratus* infection while the pathogen could effectively attack mushrooms of the species through cuts (Gea et al. 2014, 2016). A recent report on infestation of *P. ostreatus* by *H. odoratus* revealed further that the bases of fruiting bodies of this species can be more sensitive against infections by the pathogen (Gea et al. 2019), similarly to our own observations on rare events of overgrowth of mushroom stipes of *P. ostreatus* (Fig. 9).

Mycelial proliferation of *P. eryngii* is hindered by *H. odoratus* and the species is attacked by the pathogen at any cultivation stage (Kim et al. 2014). Differently to the fruiting bodies, mycelium of *A. bisporus* has been reported to be resistant e.g. for the wet bubble disease inducer *H. perniciosus* (Zhang et al. 2017) while the dry bubble inducer *L. fungicola* has variably been found to attack or not attack host mycelium (Dragt et al. 1996; Calonje et al. 2000; Shamshad et al. 2009) and the cobweb inducer *Cladobotryum varium* overgrow with time cultures of the basidiomycete and caused necrosis (Gray and Morgan-Jones 1981). Furthermore shown in this study, in mycelial confrontations with growing or grown *A. xanthodermus* and *P.* *ostreatus* cultures, the five *H. odoratus* isolates here were not or not very aggressive with both species. In contrast, the strains more strongly attacked mycelial *C. cinerea* colonies. This latter species is a dung fungus that likes higher temperatures around 37 °C best for growth (Kües 2000) while it is poorly growing at lower temperature ranges such as RT (Fig. 9). Strains of the temperate species *H. odoratus* grow in temperature ranges of 5 to 25 °C and only very poorly at warmer temperatures up to 28 °C (Back et al. 2012b). As seen in Fig. 3, strains of *H. odoratus* proliferate in nature from soil, plant litter and former mushroom residues onto their hosts. The two fungi *C. cinerea* and *H.* *odoratus* may live under quite different environmental circumstances and ecological niches why a species like *C. cinerea* with higher temperature preferences might not have developed a mycelial growth resistance at lower temperature towards this particular pathogen. As also seen in this study, *H. odoratus* does not generally infect all Agarics (Fig. 5) although the mycopathogen has an apparent preference for them. Other parasitic *Hypomyces* species appear to preferentially attack polypores and boletes (Rogerson and Samuels 1989, 1993). The broader host range is one criterium to distinguish *Hypomyces* species, temperature preferences another. *H. odoratus* and the also agaricicolous *H. rosellus* are adapted to temperate regions, whereas other *Hypomyces* species are found on mushrooms in the tropics and subtropics (Põldmaa 2011; Tamm and Põldmaa 2013). *C. cinerea* is an edible mushroom cultivated in some tropical countries including Thailand (Kües et al. 2007) and it could be of interest to test whether the species is better resistant against tropical *Hypomyces* species.

## Data Availability

Not applicable.
